# Topological Strata of Weighted Complex Networks

**DOI:** 10.1371/journal.pone.0066506

**Published:** 2013-06-21

**Authors:** Giovanni Petri, Martina Scolamiero, Irene Donato, Francesco Vaccarino

**Affiliations:** 1 ISI Foundation, Torino, Italy; 2 Dipartimento di Ingegneria Gestionale e della Produzione, Politecnico di Torino, Torino, Italy; 3 Dipartimento di Scienze Matematiche, Politecnico di Torino, Torino, Italy; University of Namur, Belgium

## Abstract

The statistical mechanical approach to complex networks is the dominant paradigm in describing natural and societal complex systems. The study of network properties, and their implications on dynamical processes, mostly focus on locally defined quantities of nodes and edges, such as node degrees, edge weights and –more recently– correlations between neighboring nodes. However, statistical methods quickly become cumbersome when dealing with many-body properties and do not capture the precise mesoscopic structure of complex networks. Here we introduce a novel method, based on persistent homology, to detect particular non-local structures, akin to *weighted holes* within the link-weight network fabric, which are invisible to existing methods. Their properties divide weighted networks in two broad classes: one is characterized by small hierarchically nested holes, while the second displays larger and longer living inhomogeneities. These classes cannot be reduced to known local or quasilocal network properties, because of the intrinsic non-locality of homological properties, and thus yield a new classification built on high order coordination patterns. Our results show that topology can provide novel insights relevant for many-body interactions in social and spatial networks. Moreover, this new method creates the first bridge between network theory and algebraic topology, which will allow to import the toolset of algebraic methods to complex systems.

## Introduction

Complex networks have become one of the prominent tools in the study of social, technological and biological systems [1]–[3]. In particular, weighted networks have been largely used to convey not only the presence but also the intensity of relations between nodes in a network. Real-world networks display however intricate patterns of redundant links with edge weights and node degrees usually ranging over various orders of magnitudes [4], [5]. This makes very hard to extract the significant network structure from the background [6]–[9], especially in the case of very dense networks [10], [11]. Alongside topological filtering methods [12], [13], the typical approach to this problem is to choose a suitable threshold for the edge weights, e.g. global [10] or local [14], and study the reduced graph composed by only the edges of weight larger (smaller) than the threshold parameter. In any case, some properties of the original graph are inevitably lost under such transformation.

To avoid this pitfall, given a weighted network 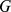 we consider the set of all filtered networks, 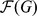, ordered by the descending thresholding weight parameter, in the spirit of *persistent homology* [15]–[18].

Persistent homology is a recent development in computational topology designed for robust shape recognition and data-discovery from high dimensional datasets [19]. It has found successful application in various fields, ranging from biological systems (e.g.brain correlation networks [20] and breast cancer diagnosis [15]), computer vision and sensor network coverage problems [15] all the way to the analysis of large scale cosmological structure [22]. Its central device is the construction of a simplicial *filtration* of the original dataset: data points are usually embedded in a metric space in order to extract from their configuration a sequence of growing simplicial complexes, which approximates with increasing precision the original dataset. Studying the changes of the topological structure along such filtration provides a natural measure of robustness for the topological features emerging across different scales. In analogy to the metric example, we call the set 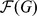 *graph filtration*: considering the set of all filtered networks captures the link weights and connectivity structure over all weight scales, without the need to resort to any assumption on an eventual metric structure underlying the graph structure. The graph filtration of a network 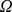 is built following these steps :

- Rank the weights of links from 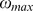 to 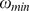: the discrete parameter 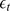 scans the sequence.
- At each step 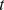 of the decreasing edge ranking we consider the thresholded graph 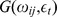, i.e. the subgraph of 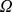 with links of weight larger than 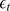.

**Figure 1:**
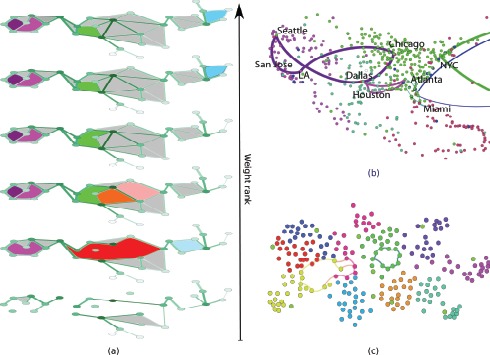
Weight rank clique filtration and homology of networks. *(a)* The weight rank filtration proceeds from the bottom up. Weighted holes (colored) and cliques (gray) appear as links are added. Weighted holes can branch into smaller holes, which have then independent evolution, persisting or dying along the filtration as links close them by 3-cliques. The cartoon shows two very long-persistence holes (violet and purple) appearing quite early and living until the end, while the largest hole (red) branches into three smaller holes, of only one survives to the end of the filtration (green). *(b)* A selection of weighted holes from the US air passenger network (year 2000). The node colors represent the best modularity partition of the entire network. The cycles are all long-persistence one, chosen to represent different behaviors: for example, the Chicago-Los Angeles-San Jose-Seattle cycle spans a large spatial distance, implying weaker connectivity across the cycle and within the region encompassed by the cycle, while the cycle going east from New York connects the east coast to three large European network and its persistence is due to the reduced connectivity due to the Atlantic Ocean. (c) A selection of the strongest cycles in the face-to-face contact network in a primary school (see SI for details on dataset). Node colors represent different classes in the school. Cycles are often found across communities, since by definition they probe the presence of holes among network regions. However, this is not the only information they convey. The cycle contained in a single community (green) testify the presence of peculiar contact geometries even within dense community structures.

Figure 1a provides a schematic illustration of the rank filtration. This approach preserves the complete topological and weight information, allowing us to focus on special mesoscopic structures: *weighted network holes*, that relate the network’s weight-degree structure to its homological backbone.

A weighted network hole of weight 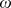 is a loop composed by 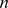 nodes 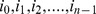, where all cyclic edges 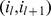 (with 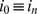) have weights 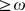, while all the other possible edges crossing the loop are strictly weaker than 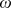. We focus on this special class of subgraphs, because formally such weighted holes represent the generators of the first homology group, 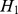, of the clique complex of the graph thresholded by weight 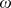 (see Materials and Methods). The aim of this paper is to characterize the evolution of these generators along the network filtration. As we swipe the network from the largest to the smallest weights, network holes appear and potentially close.

By unearthing their properties, we obtain the main contribution of this paper: the statistical features of weighted network holes yield a classification of real-world networks in two classes, depending on the compatibility or lack thereof with null models generated by graph randomisations. Furthermore, this classification is defined by mesoscopic homological structures that cannot be reconduced to local properties alone.

The method used for the classification itself, which we call *weighted clique rank homology*, is the second novel main contribution of this paper. It allows to recover complete and accurate long-range information from noisy redundant network data, by building on persistent homology [16], a recent theory developed in computational topology [17], which we extend to the case of networks.

Each weighted hole 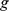 is characterized by three quantities: its birth index 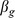, its persistence 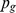 and its length 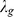. After ranking links in a descending order according to their weights, the birth index of a hole is the rank 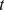 of its weight 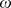. As we proceed adding links to the filtration in ranking order, it is possible that a link with rank 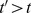 will appear and cross the hole. We call this closure of the weighted hole, or *death* 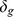. The persistence 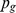 is the interval between the birth and death of 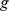, 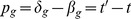. Finally, the length 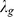 is the number of links composing 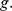


Similarly to stratigraphy, each step of the filtration is a topological stratum of the network, where the edge weight rank plays the role of depth. Intuitively, 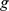 can then be thought as an underground cavity, hidden in the link-weight fabric of the network, and 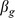, 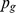 and 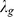 as its maximal depth, vertical size and girth respectively.

## Results

### Homological Network Classes

We applied this analysis to various social, infrastructural and biological networks (see SI for a detailed list). In order to compare datasets, indices are normalized by the corresponding filtration length (maximal rank) 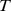, so that all 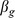, 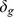, and thus 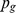, vary in the unit interval. In addition, we compared each dataset with two randomized versions, obtained by weight reshuffling and edge-swapping respectively. While both randomisations preserve the weight and degree sequences (and the relative distributions (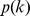 and 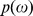), the first one redistributes only the edge weights and is meant to destroy weight correlations, preserving the joint degree distribution 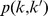 and thus the degree assortativity. The second instead randomizes the network through double-edge swaps, preserving 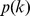 and 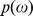 but destroying both weight and degree correlations [23]. We stress that, as the degree and weight sequences are preserved in the randomisations, they cannot account for the differences in the observed homology.

**Figure 2:**
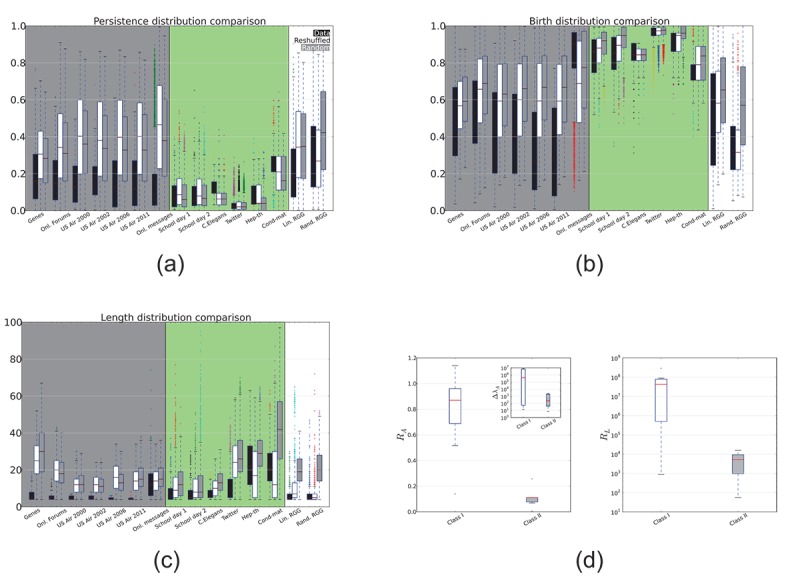
Statistical and spectral properties of 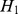 generators. Box plots of the distributions of persistences 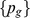 (panel 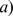), births 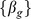 (panel 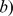) and lengths 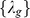 (panel 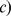) for the 1d cycles (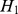 generators) of real networks (*black*), reshuffled (*white*) and randomized (*gray*). The gray and green shaded areas identify the two network classes described in the main text: class I is significantly different from the random expectations, with shorter, less persistent cycles that appear across the entire filtration; class II networks are not significantly different from the random versions, with long cycles and late birth times in the filtration. The characteristics of class I networks imply a stratification of cycles that betrays the presence of large, non-local organisation in the network structure, which is not present in class II networks. For comparison, an example of RGG network (600 nodes in the unitary disk, linking distance 0.01), known to have higher order degree correlations, had edge weights set according to 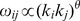, with 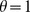 (linearly correlated weight RGG) and 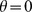 (random weight RGG). In both cases, the distributions of cycles’ properties resemble closely those of class I networks. Panel 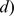 finally reports the distribution of adjacency spectral gaps 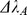 and 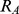 (left plot) and the Laplacian eigenratio 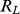 (right plot). All the quantities show significant (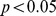) differences between the two classes, implying that the homological structure affect the dynamical properties of networks, e.g. the synchronizability threshold.

The statistical distributions obtained for the 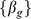, 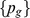 and 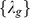 for 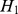 cycles highlight a natural division of the analysed networks in two broad classes (Fig. 2):

#### Class I networks

cycle distributions are markedly different from the randomized versions (cycles display shorter persistence times, earlier and broader birth distributions and very short lengths as compared to their randomized versions);

#### Class II networks

cycle distributions are very close to their random versions (late appearance, short persistences, long cycles).

The short cycles of Class I networks nest hierarchically and appear and die over all scales while those in the randomized counterparts are born uniformly along the filtration but are more persistent, producing largely hollow network instances. The implications are twofold. Since cycles represent weaker connectivity regions, this results in class I networks being more *solid* than the randomized versions, while class II networks resemble more closely the randomized instances. Second, since the cycle abundance ratio between real and random instances is the same in the two groups, the differences between class I and II does not depend on cycle abundance, but rather on their properties.

This can be seen easily by compressing the whole information within two scalar metrics which do not depend on the number of generators in a given network filtration. We define the *network hollowness* 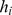 and the *chain-length normalized hollowness* 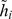 as:
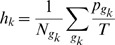
(1)

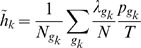
(2)where 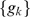 is the set of generators of the *k*-th homological group 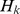 and 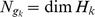 their number. The first is a measure of the average generator persistence, while the second weights generators according to both their length and persistence. Table 1 reports the values for 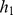 and 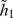. Class I networks have lower hollowness values as compared to their randomized versions, while class II ones show comparable values.

**Table 2:** Summary of spectral quantities values.

Dataset (class)	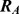	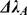	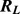
Genes(I)	1.14	14.6	873
Online forums(I)	0.5	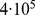	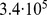
US Air 2000(I)	0.868	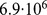	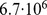
US Air 2002(I)	0.872	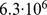	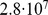
US Air 2006 (I)	0.958	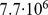	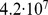
US Air 20011(I)	0.941	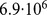	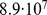
Online messages(I)	0.14	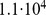	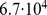
School day 1 (II)	0.11	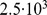	56
School day 2 (II	0.08	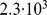	110
C. elegans (II)	0.25	76	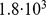
Twitter (II)	0.11	370	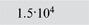
Hep-th (II)	0.11	7.4	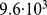
Cond-mat (II)	0.005	0.24	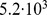
Lin. RGG	0.0034	34	836
Ran. RGG	0.018	54	255

**Summary of spectral quantities.** For each dataset, we report the values of 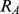, 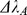 and 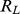. The two classes inline different spectral properties, with particular reference to 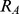 which is related to the network expansion property.

Interestingly, the hollowness values for the 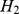 generators mostly vanish for the randomized instances (Table 1), as opposed to the case of real networks. It appears that, while persistent one-dimensional cycles are more easily generated in the randomized instances, higher forms of network coordination, e.g. 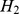 generators (akin to two-dimensional surfaces bounding three-dimensional voids), do not only display different properties in comparison to the real network, but are instead wiped away. These findings hint therefore to the presence of higher order coordination mechanisms in real world networks.

**Table 1:** Summary of hollowness values.

Dataset (class)	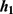	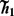	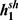	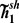	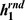	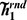	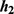	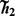
Genes(I)	0.515	0.003	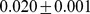	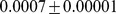	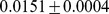	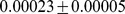	0.35	0.006
Online forums(I)	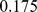	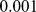	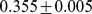	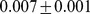	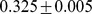	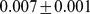	0.02	0.0003
US Air 2000(I)	0.160	0.001	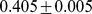	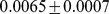	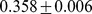	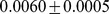	0.02	0.0003
US Air 2002(I)	0.186	0.0008	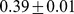	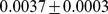	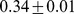	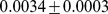	0.23	0.002
US Air 2006 (I)	0.167	0.0005	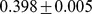	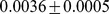	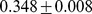	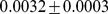	0.165	0.001
US Air 20011(I)	0.181	0.0006	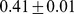	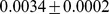	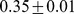	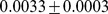	0.076	0.0007
Online messages(I)	0.21	0.0014	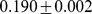	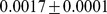	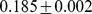	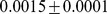	0.02	0.0003
School day 1 (II)	0.088	0.0034	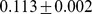	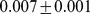	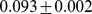	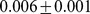	0.015	0.0012
School day 2 (II)	0.090	0.0033	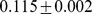	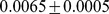	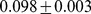	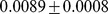	0.01412	0.00095
C. elegans (II)	0.0784	0.002	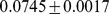	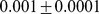	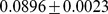	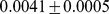	0.058	0.002
Twitter (II)	0.03	0.0001	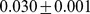	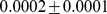	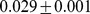	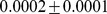	0.01	0.0001
Hep-th (II)	0.08	0.0002	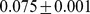	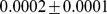	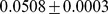	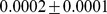	–	–
Cond-mat (II)	0.26	0.0004	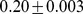	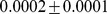	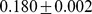	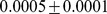	–	–
Lin. RGG	0.227	0.003	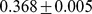	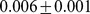	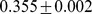	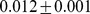	0.28	0.006
Ran. RGG	0.3	0.0041	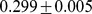	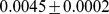	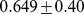	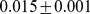	0.115	0.003

**Summary of hollowness values.** For each dataset, we report the values of the *hollowness* 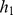 and *cycle-length normalized hollowness* 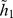 for 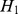 cycles for real networks and their randomisations (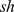 and 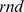). Most networks (class I in particular) show lower values than for their randomized versions. We also report the values of the *hollowness* 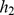 and *cycle-length normalized hollowness* 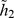 for 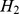 cycles for real networks. The values for the randomized networks are not reported as –strikingly– the randomisations do not inline any higher homology, while almost all real networks inline positive values of the 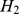 hollowness.

Naturally, the two network classes do not represent a binary taxonomy and should be considered as two extremes of a range over which networks are distributed. For example, we find networks that interpolate between these classes, e.g. the online messages network has short persistence intervals, but also late cycle appearances and short length cycles. However, classes do not appear to display uniform behavior for local and two-body quantities: degree- and weight-distributions and correlations are mixed within the same group and do not provide a direct answer for the nature of the two classes. Similarly, a recently proposed measure of structural organisation, *integrativeness* [24], which measures the neighborhood overlap around strong links, does not provide insights to explain class I, since within the latter one finds both integrative and dispersive networks.

Finally, the classes do not show a consistent pattern in *assortativity*: for example, class I includes the gene network (assortative) and the airport networks (disassortative), while class II includes the assortative co-authorship networks and the disassortative Twitter data. Therefore, assortativity cannot be the discriminating factor between classes.

### Higher Order Organization

Because homology is essentially a non-local property, it was expectable that the local measures mentioned would not be able to explain the observed homological patterns. Network homology can be seen in fact as the weighted complement to the *perturbative* 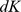-series approach [8]: the latter proceeds by successive bottom-up constraints on 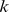-body correlations, rapidly becoming very cumbersome, while our method returns the complete superposition of the network’s degree and weight correlation layers in a non-perturbative (top-down) fashion.

A simple artificial network helps illustrating this point: Random Geometric Graphs (RGG) have been recently shown to display long-range many-body correlations [25], [26]. We find also that they have homological structures reminding of class I networks (Fig. 2a, b and c) and the same relation to their randomized versions. Class I networks are the result of high-order coordination in a similar way. This is supported also by the presence in real networks and RGGs of higher homology generators, which require elaborate coordination patterns in order to appear. While these cycles almost disappear in randomized versions of real-world networks, they are present in the case of RGGs.

For the latter and the airports, this organisation can be thought as the result of the non-local constraint imposed by the metric of the underlying space [27]. Although spatial constraints are harder to fathom for social and genetic systems, alternative explanations are possible: for example, the homological structure of the observed online communication and gene networks can be thought as stemming from group interactions among people (e.g. mailing lists, multi-user mails) and biological functions (e.g. pathways ) respectively, which provide an underlying non-local mechanism for the emergence of homological patterns.

Further evidence of this behavior can be found by zooming on specific cycles which convey information about underlying constrains hidden in the network weight-link connectivity patterns. For example, the cycle structure of the air passenger network detects the expected reduced connectivity over oceans in the form of strong persistent cycles– and the strong backbone of US airport hubs, which is then filled by the local (intra-community) links (Fig. 1b). Another example can be found in the school children’s face-to-face contact network. As expected we find the most significant cycles to link together different school classes (yellow and pink cycles in Fig. 1c). However, we also find that a school class (green nodes), despite being both a network community and 3-clique component [28], is characterized by a strong internal 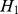 generator, which might be reflecting peculiar social dynamics coming from same-gender biases, different seating arrangements or schedules for part of the class [29].

### Spectral Correlates of Homology Classes

At the opposite extreme of local quantities lie the spectral properties of networks. It is very important therefore to investigate whether it is possible to highlight peculiar spectral signatures of the two classes. Network eigenvalues, especially those of the Laplacian matrix, figure prominently in a number of applications, ranging from spectral clustering [30] to the propensity to synchronize of a set of oscillators distributed on the nodes [31]. Given a graph 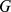, we denote its adjacency matrix 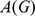 and its Laplacian matrix as 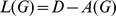, where 
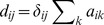
. For a symmetric network with 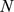 nodes, 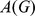 has a set of real eigenvalues 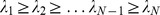. The spectral gap 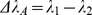, and its normalized version, 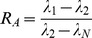, effectively measure how far the leading eigenvalue lies in comparison to the bulk of the eigenvalue distribution [32].

Interestingly, we find that class I networks have significantly larger spectral gaps (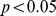 comparing the distributions) than class II networks (in Fig. 2d and Table 2 for information on individual datasets). Despite being somewhat neglected in the complex networks literature, 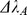 has been linked to the notion of natural connectivity [33]: it encodes spectral information about network redundancy in terms of the number of closed paths and is defined as 
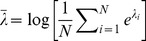
. Rewriting 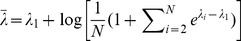, it is easy to see that for large gaps all the terms in the sum are exponentially suppressed and therefore 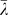 is essentially dominated by the leading adjacency eigenvalue modulo a size effect, 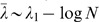. This result is consistent with the nested cycle structure that we highlighted in class I. More importantly, we find a difference between the two classes in the topological constraints to synchronization processes. For the Laplacian 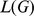, label the set of eigenvalues 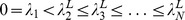 and define the Laplacian eigenratio 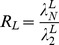. Barahona and Pecora [34] showed that a set of dynamical systems, placed on the network’s nodes and coupled according to the graph adjacency with a global coupling 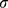, has a linearly stable synchronous state if



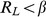
(3)where 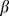 is a purely dynamical parameter. This inequality implies that networks displaying very large 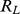 are hard (or impossible) to synchronize. Panel IVb of Fig. 2 shows again a significant difference between the two classes: class I networks have much larger eigenratios, making them hardly synchronizable.

Our results show therefore a deep connection between the homological network structure, the network spectral properties and their implications on network dynamics. Indeed, the role of mesoscopic structures in the stability and evolution of dynamical systems on networks is gradually emerging, as shown for example by recent work based on the concepts of basic symmetric subgraphs and their legacy eigenvalues in the global network spectrum [35], and is indeed being shaped by algebraic methods, well suited to capture the geometric information hidden within the network fabric.

### Conclusions

Hitherto, the homological structure of weighted networks could not be systematically studied. Our method, grounded in computational topology, allows to probe multiple layers of organized structure. It highlighted two classes of network distinguished by their homological features, which we interpreted as caused by differences in the higher order networks organisations that are not captured by (quasi)local approaches.

Among the many possible applications, two very relevant ones for social and infrastructural networks are the study of the weighted rich club’s geometry beyond the aggregate measure [23], [36], and the generalisation of network embedding models to include homological information [37]. Furthermore, the two classes displayed also a marked difference in their spectral gap distributions and in particular in the values of the algebraic connectivity, implying that the different homological structures are correlated with different synchronizability thresholds.

This work therefore provides a stepping stone towards understanding the coupling between network dynamical processes and the network’s homology.

Finally, the filtration’s construction rule is flexible and can be readily adapted to other problems. Similarly to changing goggles, different edge metrics can be used (e.g. betweenness or salience [38]), the thresholding method varied (e.g. local thresholding [14]) or the filtration promoted to a filtering on two quantities (e.g. edge weight and time in a temporal network) using *multi-persistent* homology [39].

## Materials and Methods

### Datasets

The dataset analysed in this paper cover a broad range of fields, spanning social, infrastructural and biological networks. Figures S1–S15 in the File S1 report the analysis for the individual datasets as opposed to the class-aggregate of figure 2.

In detail, they are:

#### US air passenger networks

The networks refer to the years 2000, 2002, 2006 and 2011. The years were chosen to provide snapshots of the air traffic situation at 4–5 years intervals, plus one extra (year 2000) just before the events of 9/11 which significantly affected the air transportation industry. The data used are publicly available from the website of the Bureau of Transportation Statistics (http://www.transtats.bts.gov/). Individual flights between airports were aggregated on routes as defined by origin and destination cities. The weight reported is the yearly aggregated passenger traffic.

#### C.Elegans

The network is available at http://cdg.columbia.edu/cdg/datasets and reports a weighted, directed representation of the C. Elegans’s neuronal network [40]. The network was symmetrized by summing the weights present on edges between the same nodes (given 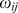 and 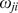, 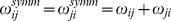).

#### Online messages and forums

The online messages network consists of messages in a student online community at University of California [41]. The online forum network refers to the same online community, but focuses on the activity of users in public forums, rather than on private messages [42]. Both networks are publicly available online at Tore Opsahl’s website (http://toreopsahl.com/datasets/).

#### Gene network

The gene interaction network used in the paper is a sampling of the complete human genome dataset available from the University of Florida Sparse Matrix Collection. Each node is an individual gene, while the edges correlates the expression level of a gene with that of the genes (using a NIR score [43]). The node set of the analysed network was obtained by randomly choosing an origin node, then adding its neighborhood to the node set; the neighborhoods of the newly added nodes were then added to the node set recursively until a given number of nodes was obtained (in the case used the target number of nodes was 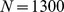). Then all the edges present in the original network between the nodes in the node set were added, effectively taking a connected subgraph of the original network. To reduce the computational complexity due to the large density of the graph, the weighted clique filtration was stopped at an edge weight of 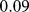 (similarly to the choice made in [24]).

#### Twitter

The dataset consists of a network of mentions and retweet between Twitter users and is available online on the Gephi dataset page (http://wiki.gephi.org/index.php/Datasets). Weights are proportional to the number of interactions between a pair of users.

#### School face-to-face contact network

The dataset contains two days of recorded face-to-face interactions in a primary school. Each node represents a child, with the edge weight between two nodes being proportional to the amount of time the two children spent face to face. We analysed the two days separately, yielding two networks. The dataset has been collected by the Sociopattern project (http://www.sociopatterns.org/) and analysed in [29].

#### Co-authorship networks

The networks analysed are the weighted co-authorship networks of the Condensed Matter E-print Archive between 1995 and 1999 (cond-mat) and the High-Energy Theory E-print Archive between 1995 and 1999 (hep-th) [44].

The graph edgelists used in the paper are available online as part of the code package we developed [45].

Finally, for comparison we use Random Geometric Graphs (RGG) [46], [47], which are simple models of spatial networks: a RGG is generated by sprinkling 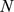 of nodes randomly on a metric space that acts as a substrate (usually a disk of unitary radius or a square with identified edges), and then linking nodes that are closer than a given linking distance 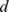.

The networks analysed in this article are undirected and weighted, because the weighted clique filtration finds a natural application in such case. However, schemes for directed networks can be easily devised and tailored to specific case studies, e.g. one could adopt the definition used in the directed clique percolation method [48] in order to associate network structures to simplices.

### Persistent Homology

The method we use to uncover weighted holes is persistent homology of the weight clique rank filtration. In this section we will briefly explain persistent homology and its realization through the weight rank clique filtration.

Persistent homology is a technique from computational algebraic topology that can be viewed as parametrized version of simplicial homology [49]. The two definitions needed for simplicial homology are those of *simplicial complex* and *homology*. A *simplicial complex* is a non empty family 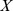 of finite subsets, called faces, of a vertex set with the two constraints:

- a subset of a face in 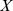 is a face in 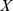,
- the intersection of any two faces in 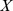 is either a face of both or empty.

We assume that the vertex set is finite and totally ordered. A face of 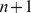 vertices is called 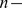
face and denoted by 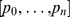. The interpretation of low dimensional faces is intuitive: a 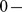
face is a vertex, a 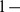
face is a segment, a 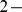
face is a full triangle, a 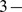
face is a full tetrahedron. The dimension of a simplicial complex is the highest dimension of the faces in the complex.

Morphism between simplicial complexes are called simplicial maps. A simplicial map is a map between simplicial complexes with the property that the image of a vertex is a vertex and the image of a 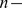
face is face of dimension 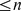.

*Simplicial Homology* with coefficients in a field is a functor from the category of simplicial complexes to the category of vector spaces [49]. Homology of dimension 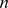 assigns to each simplicial complex 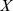, the vector space 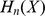 of 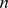-cycles modulo boundaries and to every simplicial map 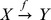 the linear map 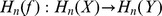.

The construction that leads to the vector space 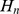 is the following. Given a simplicial complex 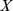 of dimension 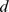, consider the vector spaces 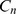 on the set of 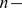
faces in 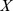 for 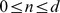. Elements in 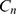 are called 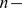
chains. The linear maps sending a 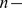
face to the alternate sum of its 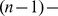
faces
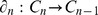


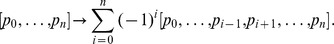
shares the property 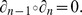


The subspace 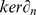 of 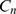 is called the vector space of 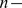
cycles and denoted by 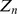. The subspace 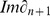 of 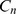, is called the vector space of 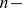
boundaries and denoted by 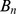. Note that from 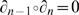 it follows that 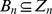 for all 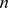.

The 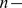
th simplicial homology group of 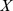, with coefficients in 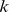, is the vector space 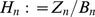.

*Persistent homology* is the homology of a *filtration*, i.e. an increasing sequence of simplicial complexes
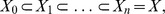
as opposed to that of a single simplicial complex.

It assigns to a filtration the homology groups of the simplicial complexes 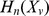 and the linear maps 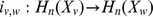 induced in homology by the inclusions 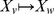 for all 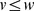. Note that the linear maps 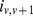 are not always injective, meaning that some homological features can disappear along the filtration. These features are encoded by the persistent homology generators: an element 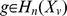 such that there is no 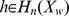 for 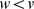 with the property that 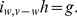 Two indices completely determine a generator 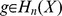, namely its birth, 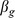 and its death 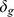. The index 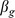 traces the first index such that 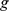 is in the filtration and 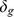 is the index of the simplicial complex in which the cycle becomes a boundary (i.e. disappears homologically). The persistence (lifetime) of a generator is measured by 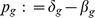. The length of a cycle, that is the number of faces composing it, is denoted by 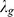.

For each homology group, the information about the filtration is collected in a barcode: the set of intervals 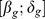 for all generators 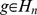, which constitutes a handy complete invariant of 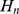 [16]. An alternative way to represent the persistent homology of a filtration is through persistence diagrams [16], [50], which we use extensively in the SI. A persistence diagram is a set of points in the plane counted with multiplicity. It can be recovered from the barcode considering the points 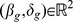 with multiplicity given by the number of generators with the same persistence interval. In the SI, the reader can find 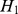 persistent diagrams of the real world datasets examined for the classification, together with the explicit comparison to the results for their relevant randomized versions.

### Filtrations

In classical applications, the filtration is obtained from a point cloud using the Rips-Vietoris complex and persistent homology used to uncover robust topological features of the point cloud. We instead use the clique weight rank filtration to uncover properties deriving from the topology and weighted structure of weighted networks.

Recalling that an 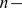
clique is a complete subgraph on 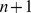 vertices, the *clique complex* is a simplicial complex built from the cliques of a graph. Namely there is a 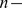
face in the simplicial complex for every 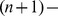
clique in the graph. The compatibility relations are satisfied because subsets of cliques and intersection of cliques are cliques themselves.

The *Weight Rank Clique filtration* on a weighted network 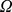 combines the clique complex construction with a thresholding on weights following three main steps.

- Rank the weights of links from 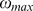 to 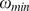: the discrete parameter 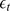 indexes the sequence.
- At each step 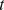 of the decreasing edge ranking we consider the thresholded graph 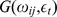, i.e. the subgraph of 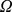 with links of weight larger than 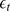.
- For each graph 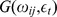 we build the clique complex 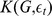.

The clique complexes are nested along the growth of 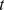 and determine the weight rank clique filtration. Note that this construction is in fact the clique complex of each element in the graph filtration.

In particular, persistent one dimensional cycles in the weight rank clique filtration represent weighted loops with much weaker internal links.

There is a conceptual difference in interpreting 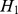 persistent homology of data with the Rips-Vietoris filtration and 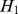 persistent homology of weighted networks with the weight rank clique filtration. While in the first case persistent generators are relevant and considered features of the data, short cycles are more interesting for networks. This is because random networks, or randomisations of real networks, display one dimensional persistent generators at all scales, while short lived generators testify the presence of local organisation properties on different scales.

### Computational Complexity

Computing the filtration of a large dataset can be extremely demanding computationally. The identification of the maximal cliques requires in general exponential time, although algorithms exists for special cases that allow solutions to be obtained in polynomial time. In addition, the javaPlex library [51] requires the explicit enumeration of the simplicial facets appearing at each filtration step, which implies the need for large memory resources in order to calculate the persistent homology. However, there are a number of simplifications and improvements to the brute force approach that provide a significant reduction of the problem’s complexity. In the metrical case, this is usually done by constructing a smaller complex, the *witness complex* [52], which approximates with controlled precision [52] the homology of the original data.

In the case of non-metrical discrete spaces, for example networks, one cannot easily construct a witness complex through a controlled sub-sampling of the network. Luckily, it is still possible to reduce the computational complexity in different ways: first, one can limit the analysis to the first 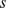 homology groups, which amounts to restricting the clique detection and storage to cliques up to size 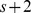, which reduces the problem to polynomial in time and memory; second, it is possible to parallelize the computation of persistent homology [53]; finally, the more elegant solution is to calculate the homology of an homologically equivalent but much smaller filtration (see the tidy set construction [54]). With respect to the standard clique complex case, the tidy set in particular was shown to reduce the number of simplices along the filtration of various orders of magnitude number of simplices and of one order of magnitude the total memory required. Therefore, a combination of the techniques mentioned above allows to scale up dataset sizes to large-scale networks.

## Supporting Information

File S1 (PDF)
