# Supplementary material for: Topological Strata of Weighted Complex Networks
Source: PLoS One. 2013 Jun 21;8(6):e66506. doi: 10.1371/journal.pone.0066506 (PMC3689815; doi:10.1371/journal.pone.0066506)
Supplement: File S1 — (PDF) [file pone.0066506.s001.pdf]

Supplementary Information for "Topological strata of weighted complex networks"

Giovanni Petri*
ISI Foundation, Via Alassio 11/c, 10126 Torino - Italy

Martina Scolamiero†
Dipartimento di Ingegneria Gestionale e della Produzione, Politecnico di Torino, C.so Duca degli Abruzzi n.24, Torino, 10129, Italy and ISI Foundation, Via Alassio 11/c, 10126 Torino - Italy

Francesco Vaccarino‡ and Irene Donato§
Dipartimento di Scienze Matematiche, Politecnico di Torino, C.so Duca degli Abruzzi n.24, Torino, 10129, Italy and ISI Foundation, Via Alassio 11/c, 10126 Torino - Italy

* giovanni.petri@isi.it
† martina.scolamiero@polito.it
‡ francesco.vaccarino@polito.it
§ irene.donato@polito.it

The Supplementary Information contains the plots illustrating the features of the H_1 generators for each analyzed dataset.

I. RESULTS FOR WEIGHT RANK CLIQUE FILTRATION

We recall that, given a network G on N nodes, we consider the weight clique rank filtration on G. Let T be the length of the filtration, $\{g_i\}$ the set of generators of the $i-$th persistence homology module of the filtration and N_{g_i} the cardinality of $\{g_i\}$. For every generator g_i, the index p_{g_i} is its persistence interval, the index λ_{g_i} is its length and β_{g_i} is its birth index. For brevity, H_1 generators will be denoted by g rather than g_1.
There is a conceptual difference in interpreting H_1 for the persistent homology of data with the Rips-Vietoris filtration and H_1 for the persistent homology of weighted networks with the weight rank clique filtration. While in the first case persistent generators are relevant and considered features of the data, short cycles are more interesting for networks. This is because random networks, or randomisations of real networks, display one-dimensional persistent generators at all scales, while short lived generators testify the presence of local organisation properties on different scales.
As stated in the main text, the complex networks we considered fall in two main groups.
Networks in group I display clear departures from the null counterparts, while class II networks show homological features that are much closer to the randomized versions. We collected the complete information about the indices p_g, λ_g and β_g for persistent H_1 generators within a series of tableaux (Figures S.1 to S.15). In every figure, panel *a*) represents the distribution of persistence p_g, panel *b*) the distribution of length λ_g and panel *c*) the distribution of birth index β_g. These quantities are studied for the homology generators in the real world network(red circles), after weight reshuffling of the network(blue squares) and in the network randomisation(green triangles). Panel *d*) is the persistence diagram of the network under study, panel *e*) is the persistence diagram of its weight reshuffled null model and panel *f*) is the persistence diagram of the random null model.
From the perspective of persistence diagrams, class I presents a rich structure of nested cycles covering all scales, as opposed to the weight reshuffled null model and random null model where generators are born uniformly along the filtration and tend to be very persistent, producing largely hollow network instances.
The degree and weight sequences are preserved in the randomisations and therefore cannot account for the differences in the homology. Another possibility to explain the different behaviour of the two classes could be the presence of degree-degree or weight-degree correlations in class I. However, networks in the two classes do not show consistent patterns of assortativity: for example, class I includes the gene network (assortative) and the airport networks (disassortative), while class II includes the assortative co-authorship networks and the disassortative Twitter data. Also weight-degree correlations do not appear to be decisive: for example, the RGGs generated with random edge weights did not show significant differences from those generated with edge weights correlated positively to the degrees of the end nodes (see Figs. S.14 and S.15).

---

[1] Carlsson, G., Topology and Data. *Bulletin of the American Mathematical Society.* **46(2)**, 255-308 (2009).

[2] Carlsson,G. & Zomorodian,A., Computing Persistent Homology. *Discrete Comput. Geom.* **33(2)**, 249-274 (2005).

[3] Edelsbrunner,H. & Harer, J., Persistent homology - a survey. *Cont. Math.*, 257-282, (2008).

[4] Munkres, J.R., Elements of Algebraic Topology *Addison-Wesley Publishing Company, Inc.*, 2725 Sand Hill Road Menlo Park, California 94025 (1984).

[5] Steiner,D.C. & Edelsbrunner, H. & Harer, J. Stability of Persistence Diagrams. *Discrete Comput. Geom.* **37(1)**, 103-120 (2007).

[6] Horak, D., Maletić, S. & Rajković, M. Persistent homology of complex networks. *J. Stat. Mech.* **2009**, P03034 (2009).

[7] Watts, D. J. & Strogatz, S. H. Collective dynamics of 'small-world' networks. *Nature* **393**, 440-442 (1998)

[8] Opsahl, T. & Panzarasa, P., Clustering in weighted networks. *Soc. Net.* **31**, (2), 155-163, (2009)

[9] Opsahl, T., Triadic closure in two-mode networks: Redefining the global and local clustering coefficients. *Soc. Net.*, (2010), 10.1016/j.socnet.2011.07.001

[10] Gardner,T.S., di Bernardo,D., Lorenz,D., & Collins,J.J., Inferring genetic networks and identifying compound mode of action via expression profiling. *Science* **301**, 102-105 (2003).

[11] Pajevic, S. & Plenz, D., The organization of strong links in complex networks. *Nat. Phys.*,**8**, 429-436 (2012).

[12] Stehlé, J. et al., High-Resolution Measurements of Face-to-Face Contact Patterns in a Primary School. *PLoS One* **6**(8): e23176 (2011)

[13] Newman,M. E. J. The structure of scientific collaboration networks, *Proc. Natl. Acad. Sci. USA* **98**, 404-409 (2001).

[14] Barthélemy, M., Spatial Networks *Phys. Rep.* **499**, 1 (2011).

[15] Penrose, M., Random Geometric Graphs *Oxford University Press*, Oxford, UK, (2003).

[16] Palla, G. et al. Directed network modules. *New J. Phys.* **9**, (2007)

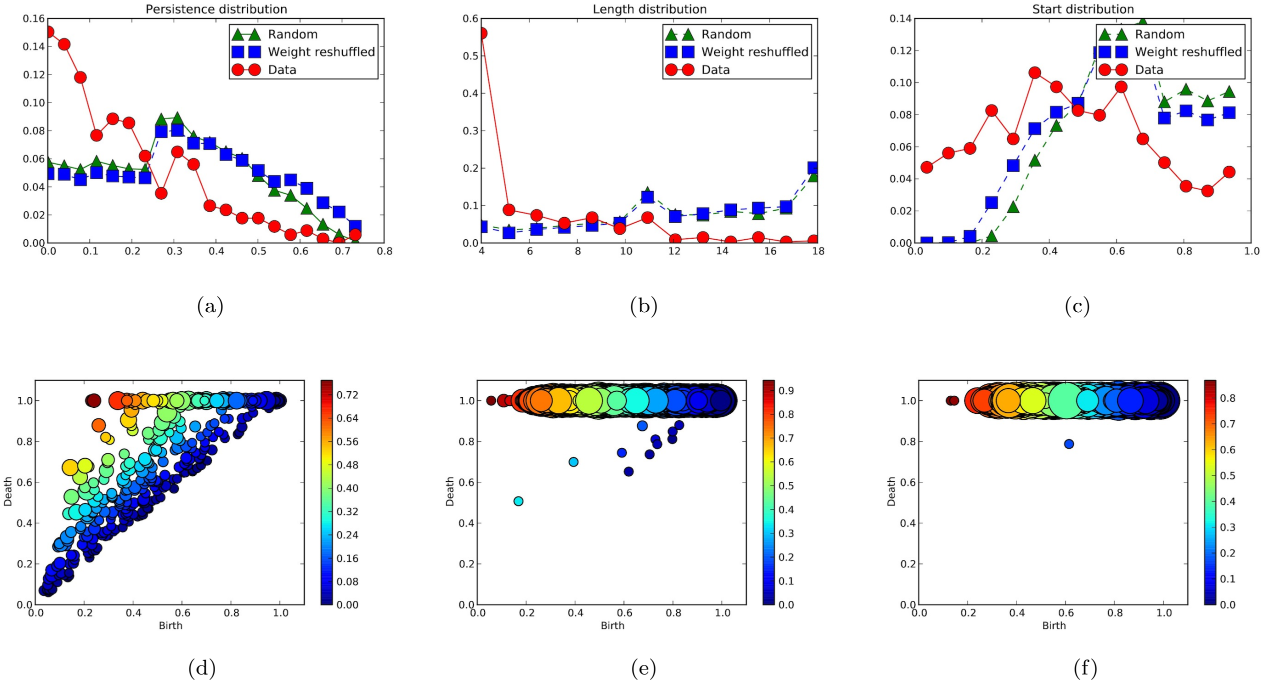

FIG. S.1: **Summary of H_1 persistent homology results for the human gene interaction network 2 (Class I).**

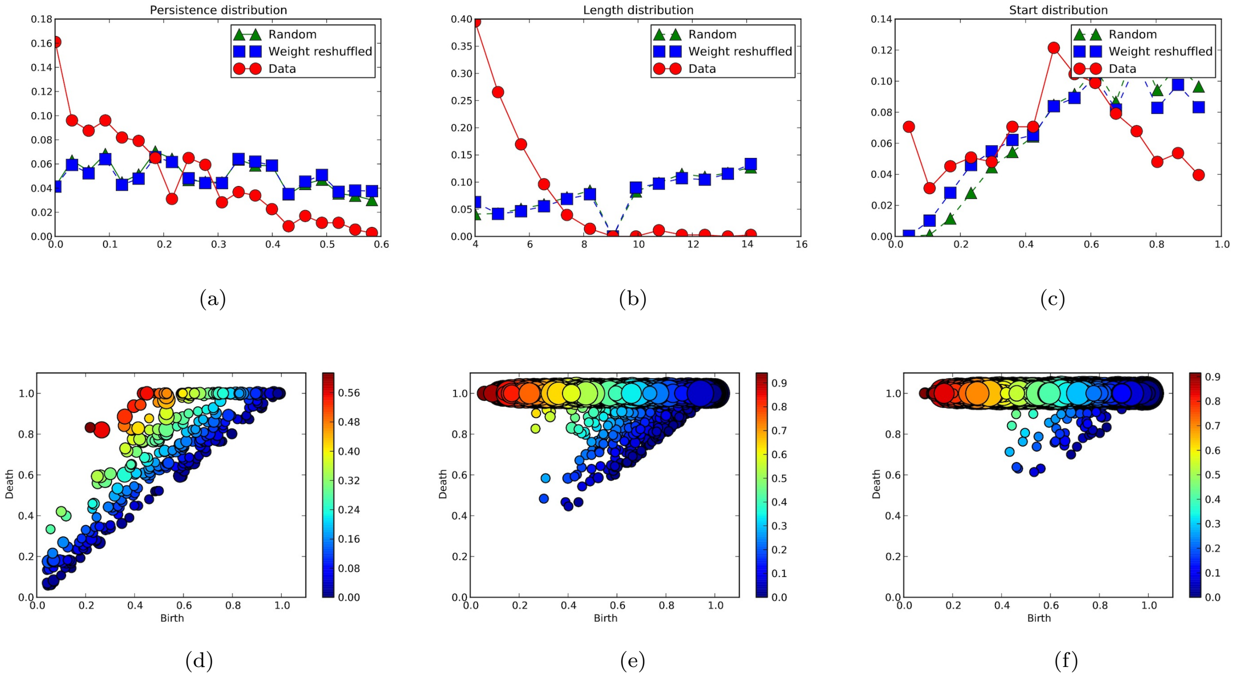

FIG. S.2: **Summary of H_1 persistent homology results for online forum network of [9] (Class I).**

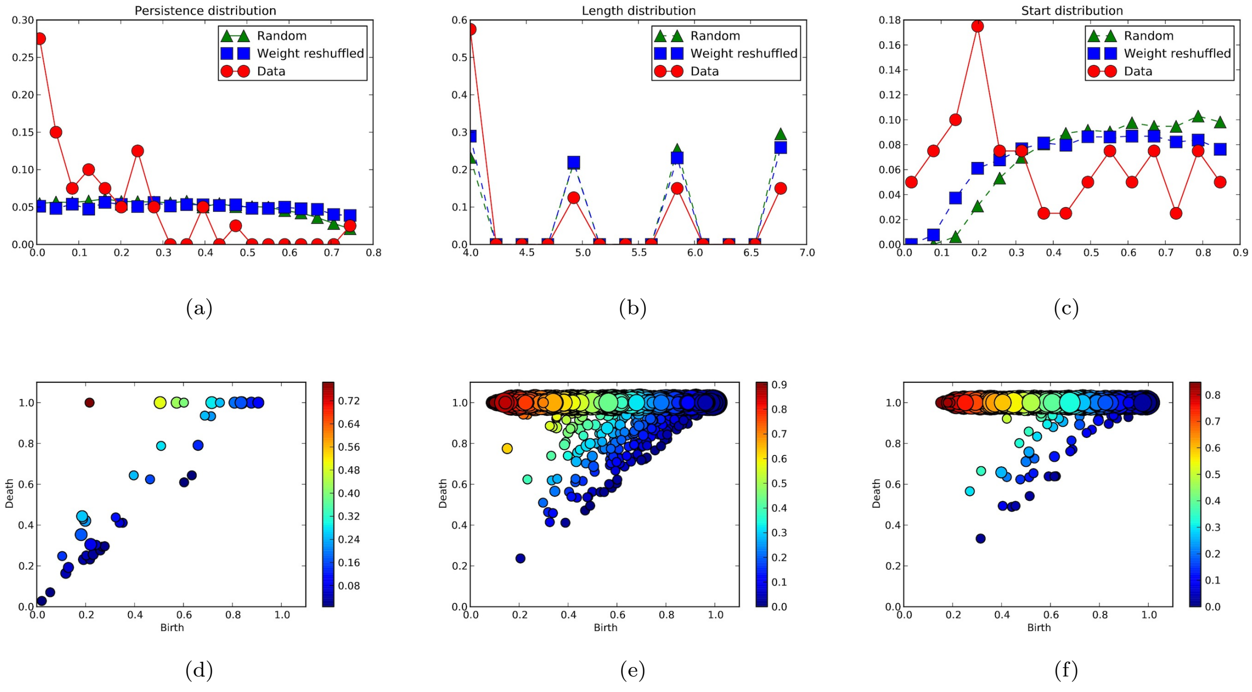

FIG. S.3: **Summary of H_1 persistent homology results for the US airways passenger network for 2000 (Class I).**

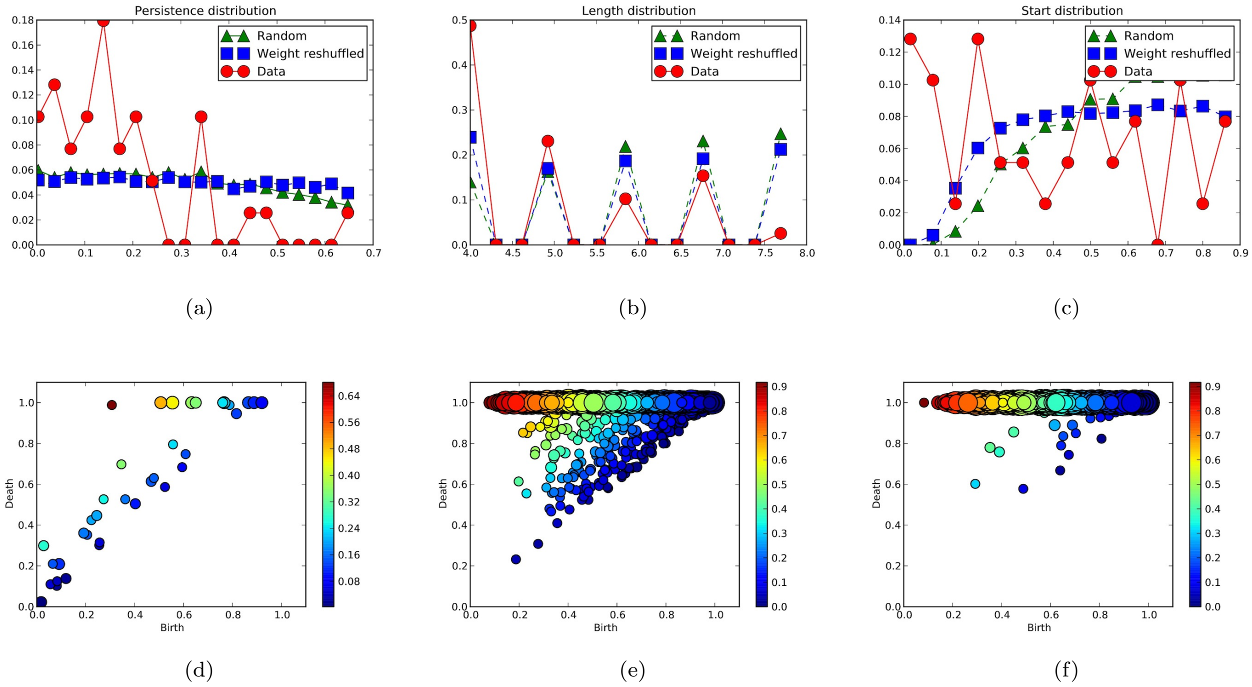

FIG. S.4: **Summary of H_1 persistent homology results for the US airways passenger network for 2002 (Class I).**

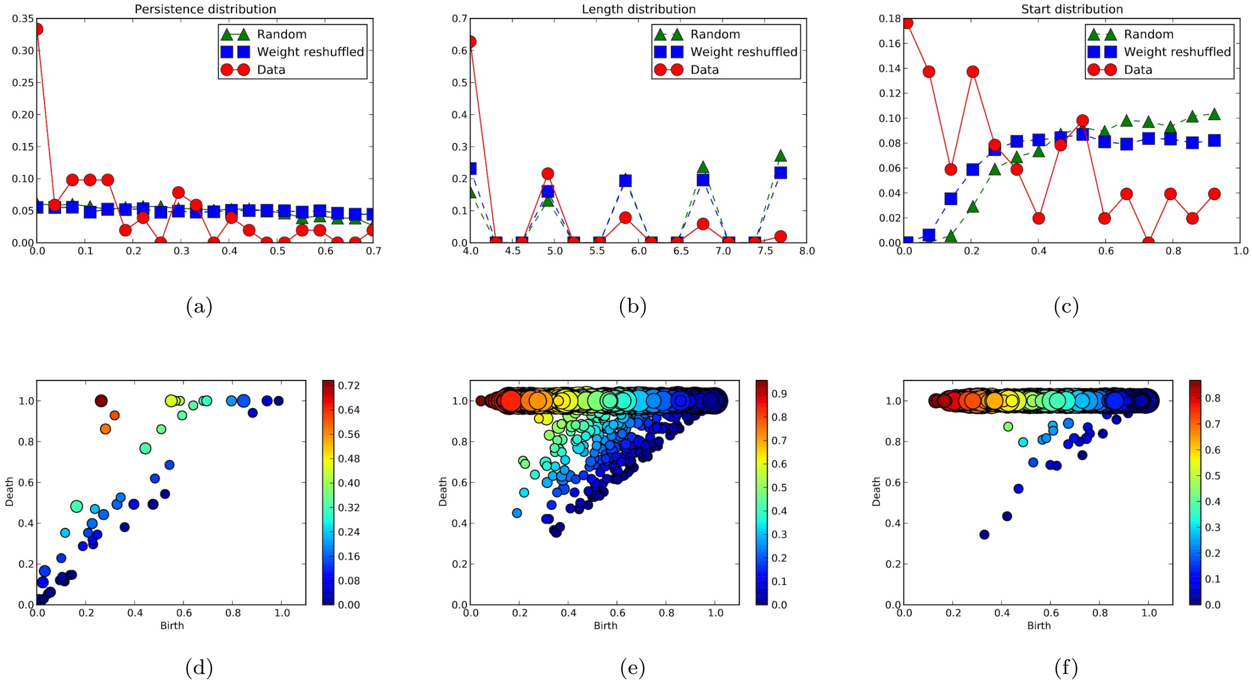

FIG. S.5: **Summary of H_1 persistent homology results for the US airways passenger network for 2006 (Class I).**

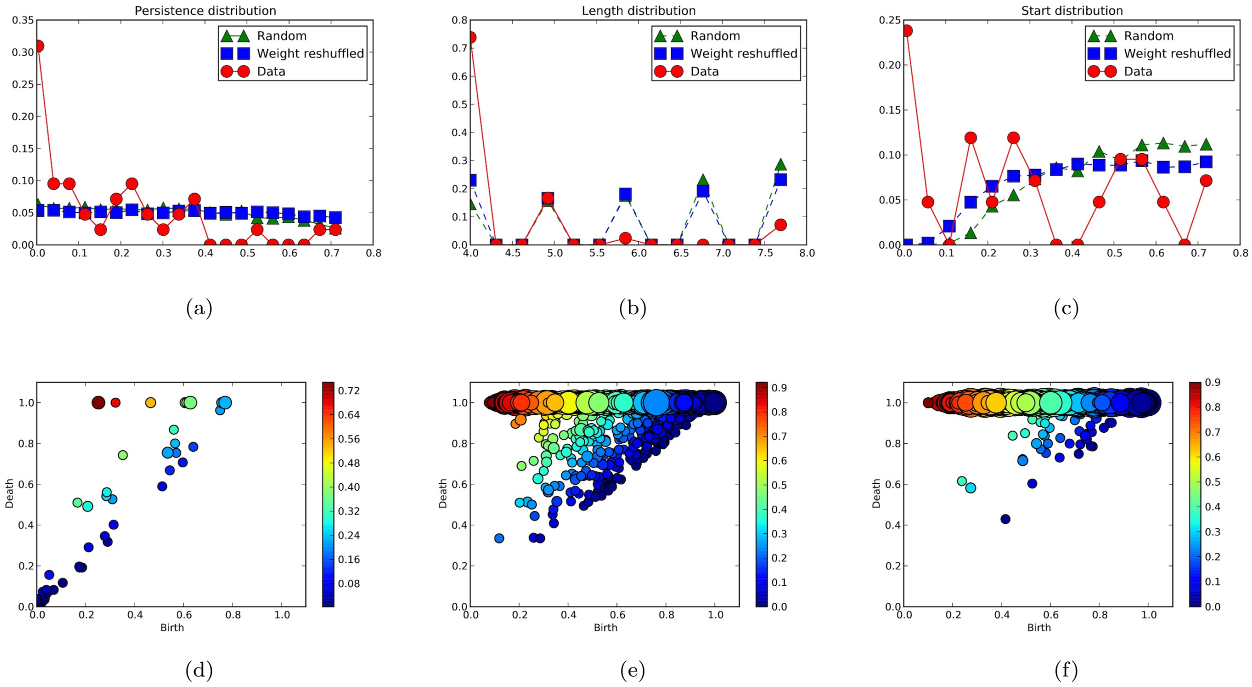

FIG. S.6: **Summary of H_1 persistent homology results for the US airways passenger network for 2011(Class I).**

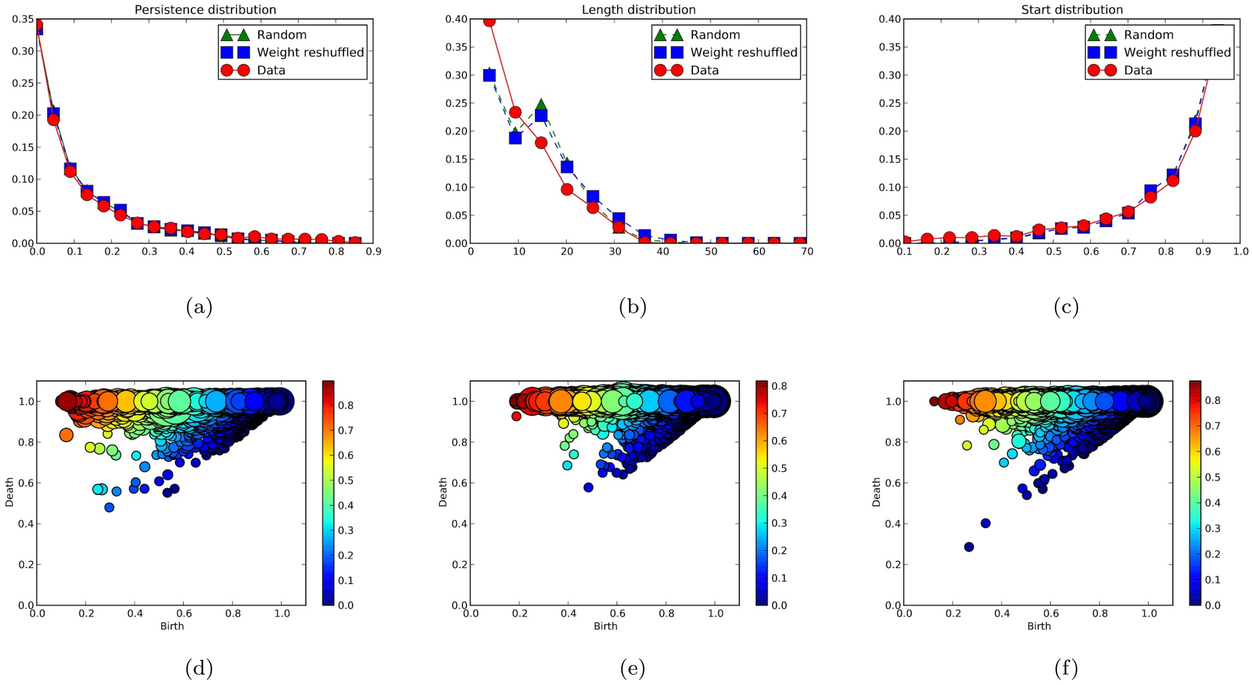

FIG. S.7: **Summary of H_1 persistent homology results for the online messages network of [8] (Class I).**

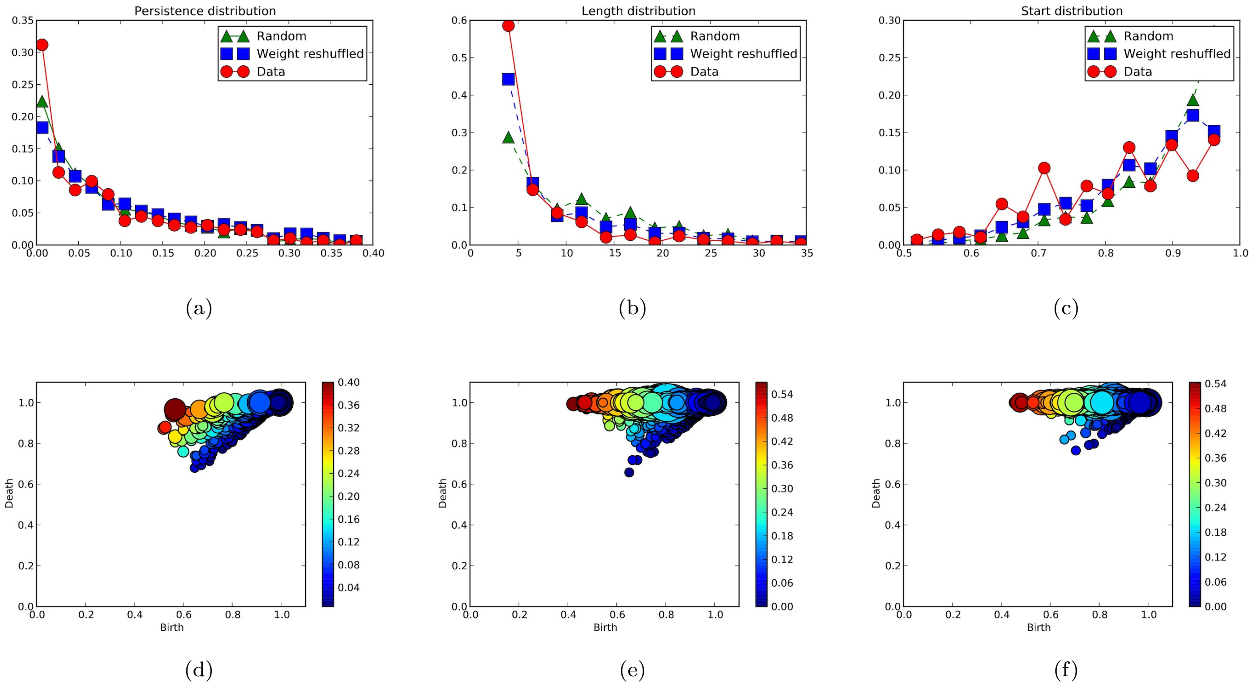

FIG. S.8: **Summary of H_1 persistent homology results for the day 1 face-to-face contact duration network of children of [12] (Class II).**

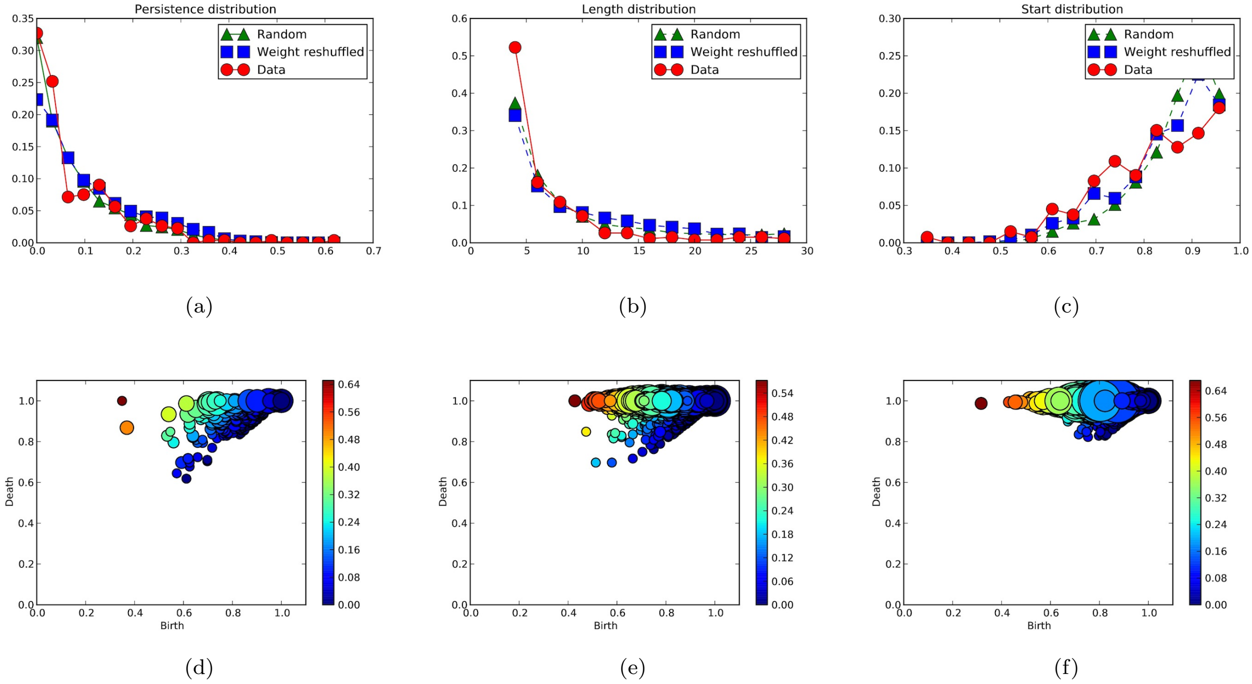

FIG. S.9: **Summary of H_1 persistent homology results for the day 2 face-to-face contact duration network of children of [12] (Class II)**

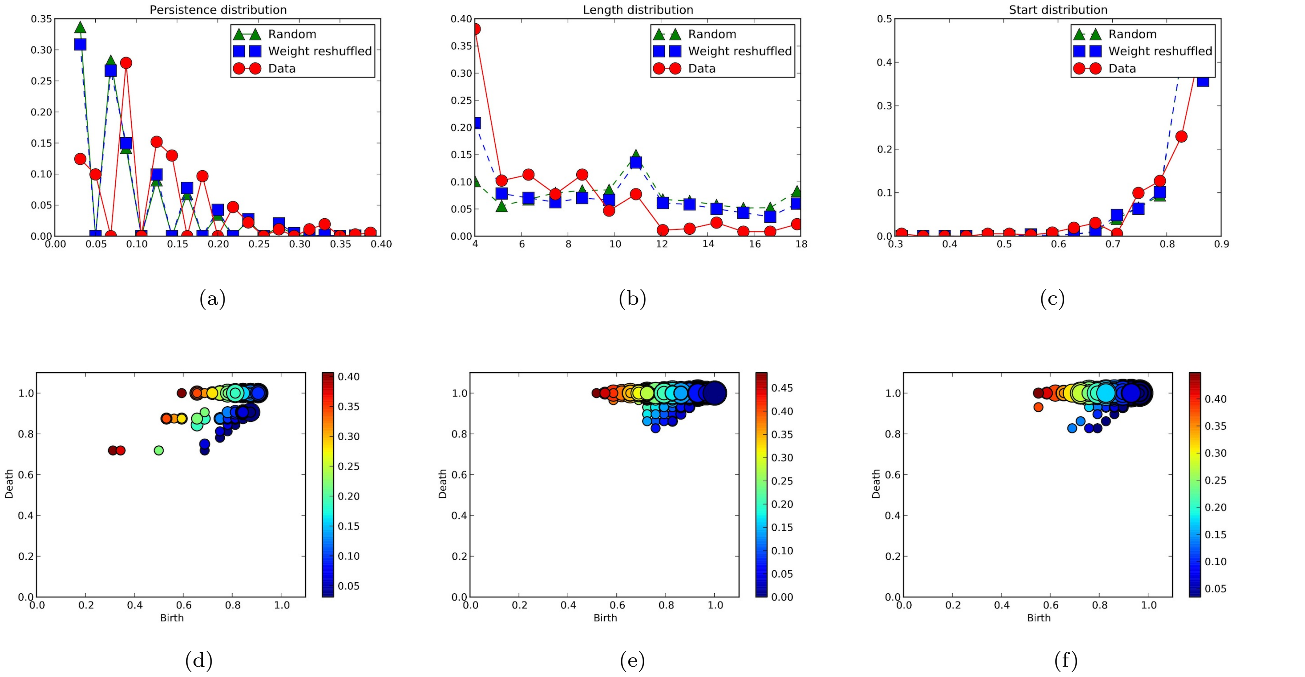

FIG. S.10: **Summary of H_1 persistent homology results for the neural network of the *C. elegans* (Class II).**

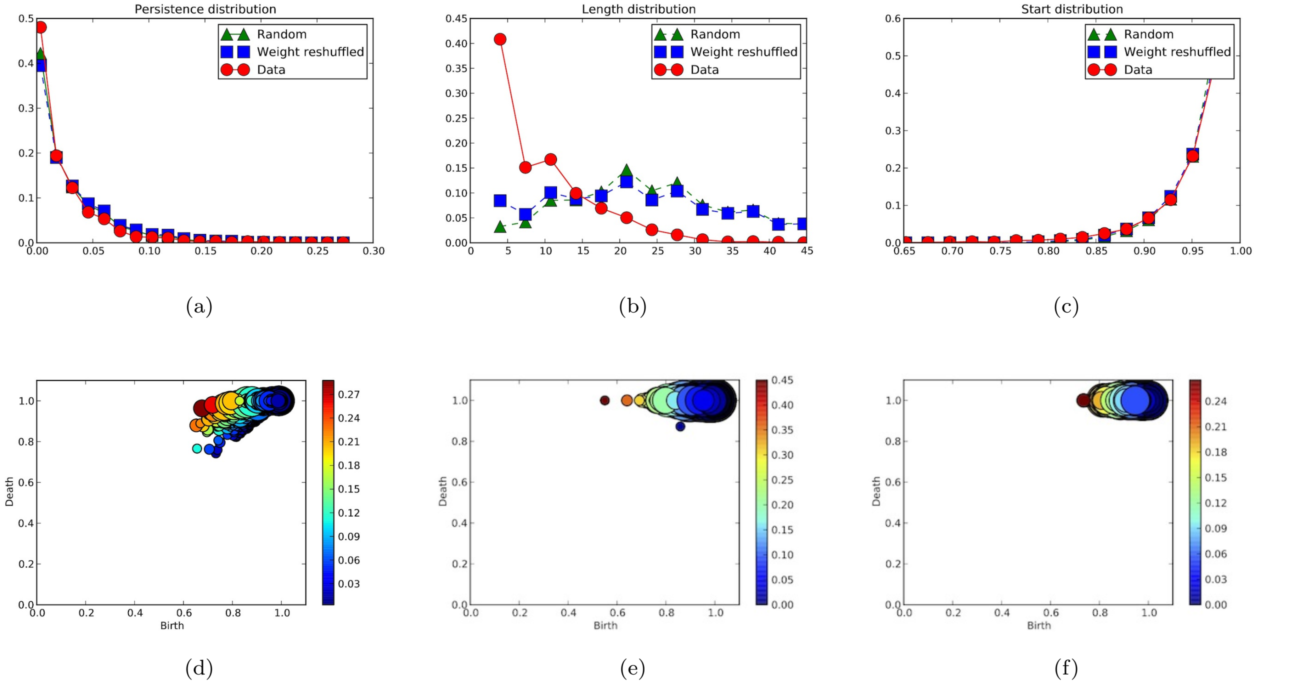

FIG. S.11: **Summary of H_1 persistent homology results for a network of mentions and retweets of a part of the Twitter network (Class II).**

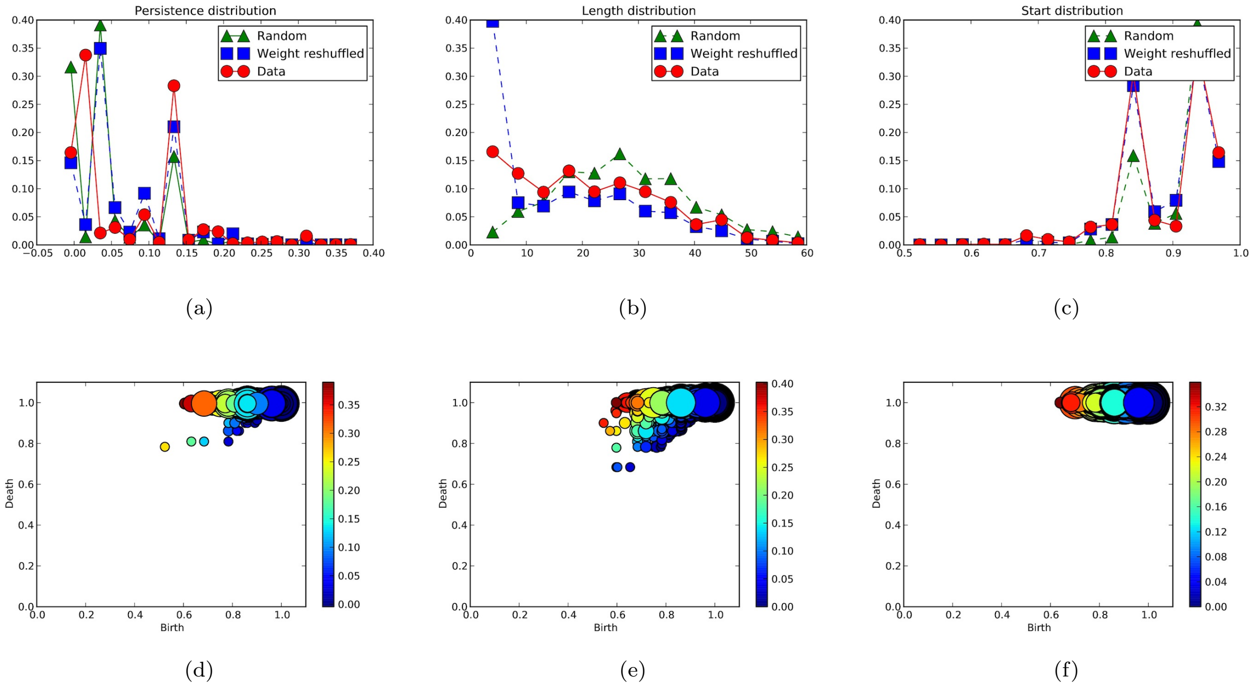

FIG. S.12: **Summary of H_1 persistent homology results for the Hep-th arxiv.....(Class II)**

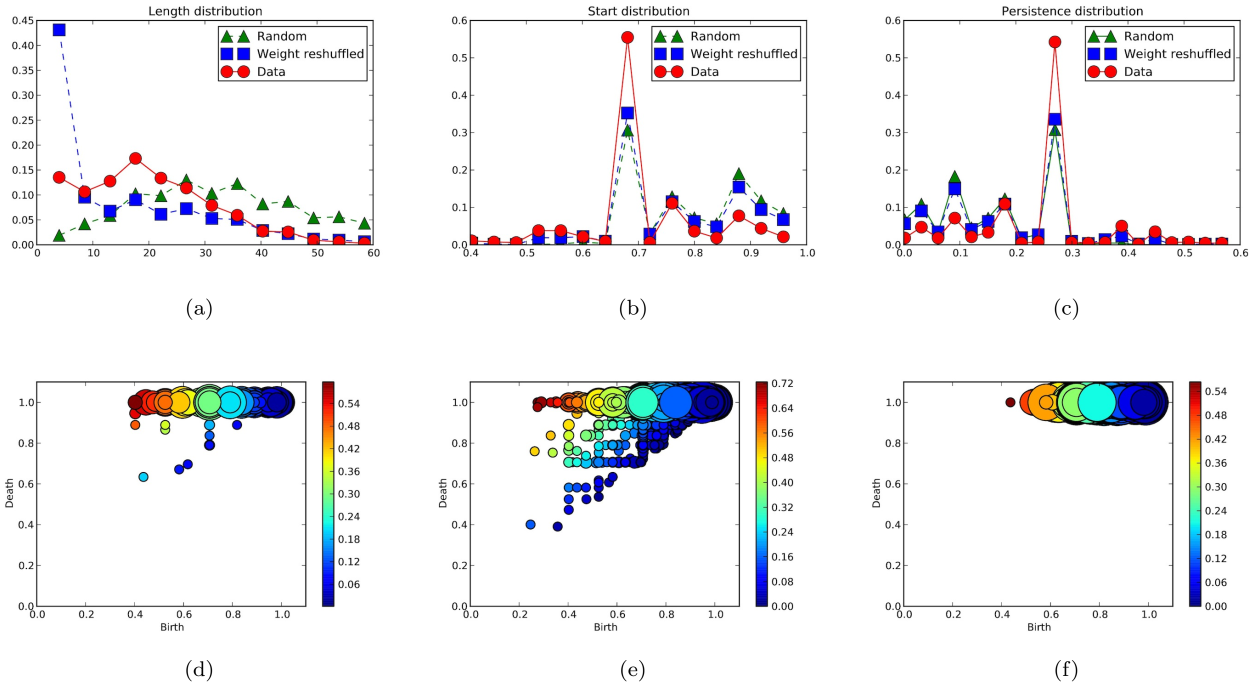

FIG. S.13: **Summary of H_1 persistent homology results for the cond-mat (Class II).**

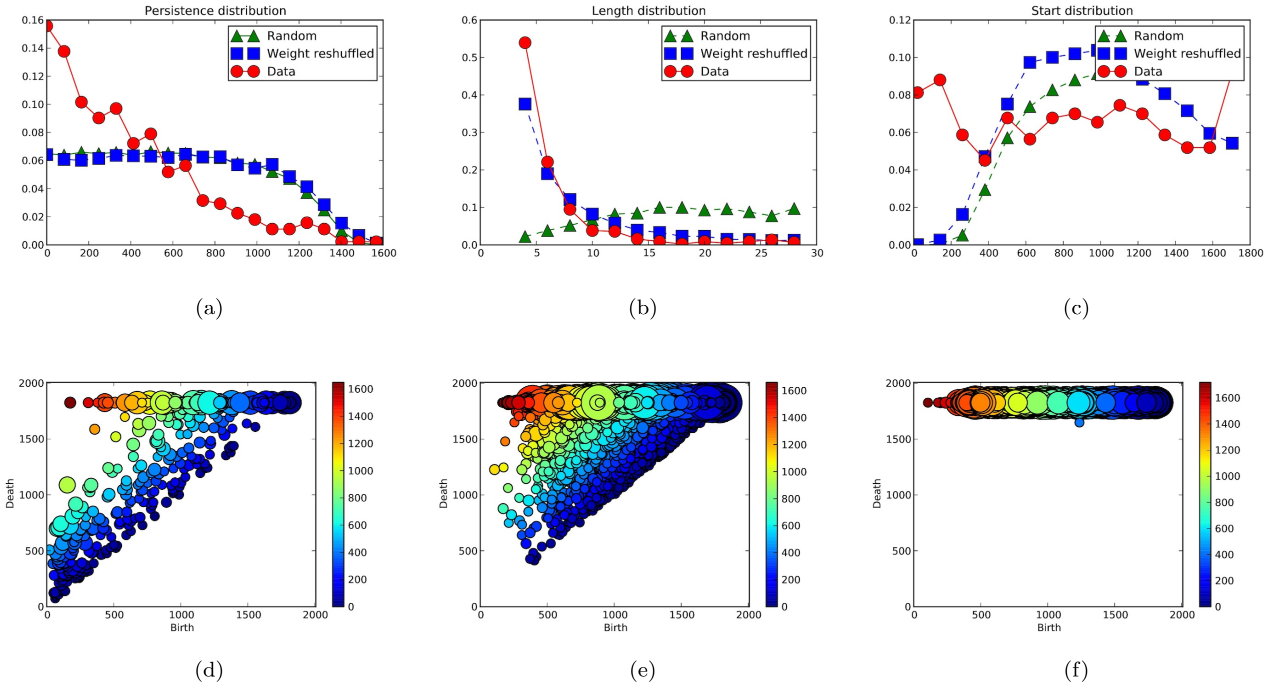

FIG. S.14: **Summary of H_1 persistent homology results for the Random Geometric Graph model with linear weight-degree correlations (Class I).** The graph has $N = 600$ nodes and a linking distance $d = 0.01$. The weight of a link between nodes i and j was set according to $\omega_{ij} \sim (k_i k_j)^{\theta} X$, where $\theta = 1$ and X is a uniform random variable in $(0, 1)$.

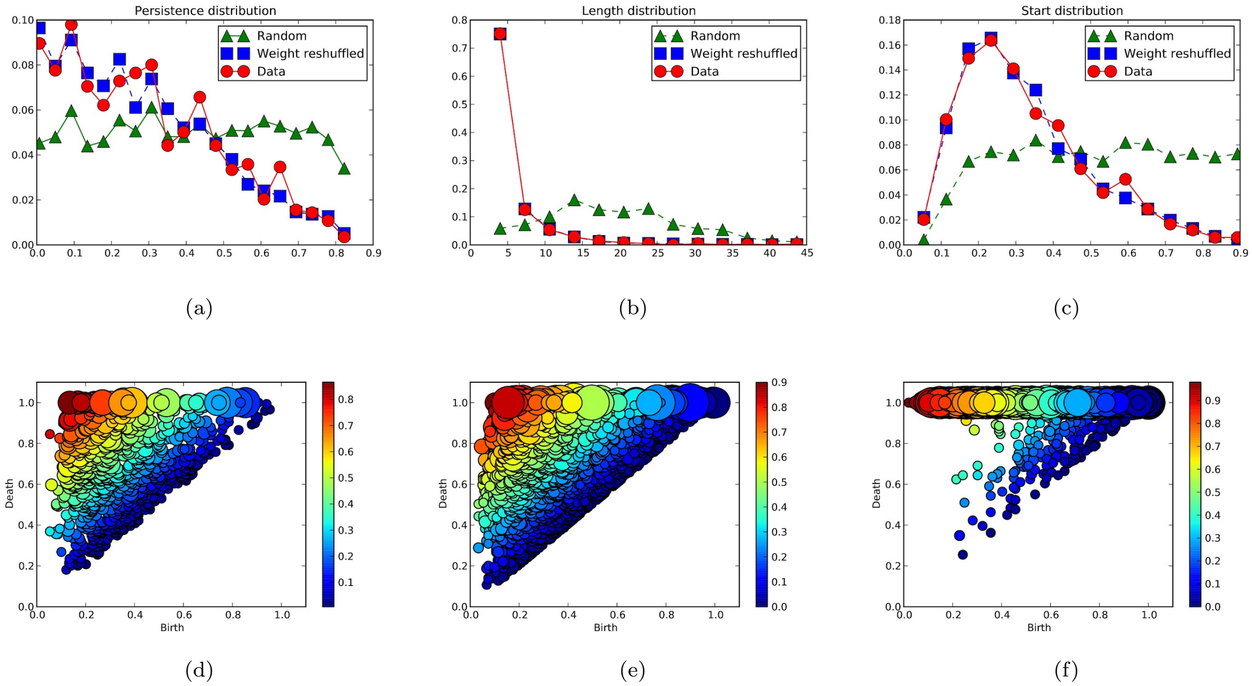

FIG. S.15: **Summary of H_1 persistent homology results for the Random Geometric Graph model with linear weight-degree correlations (Class I).** The graph has $N = 600$ nodes and a linking distance $d = 0.01$. The weight of a link between nodes i and j was set with random uniform weights .
